# The Microbial Connection to Sustainable Agriculture

**DOI:** 10.3390/plants12122307

**Published:** 2023-06-14

**Authors:** Kalaivani Nadarajah, Nur Sabrina Natasha Abdul Rahman

**Affiliations:** Department of Biological Sciences and Biotechnology, Faculty of Sciences and Technology, University Kebangsaan Malaysia, Bangi 43600, Malaysia; nursabrinatasha@gmail.com

**Keywords:** microbiome, biofertilizer, biocontrols, beneficial organisms, microbiome engineering, multidisciplinary omics technologies

## Abstract

Microorganisms are an important element in modeling sustainable agriculture. Their role in soil fertility and health is crucial in maintaining plants’ growth, development, and yield. Further, microorganisms impact agriculture negatively through disease and emerging diseases. Deciphering the extensive functionality and structural diversity within the plant–soil microbiome is necessary to effectively deploy these organisms in sustainable agriculture. Although both the plant and soil microbiome have been studied over the decades, the efficiency of translating the laboratory and greenhouse findings to the field is largely dependent on the ability of the inoculants or beneficial microorganisms to colonize the soil and maintain stability in the ecosystem. Further, the plant and its environment are two variables that influence the plant and soil microbiome’s diversity and structure. Thus, in recent years, researchers have looked into microbiome engineering that would enable them to modify the microbial communities in order to increase the efficiency and effectiveness of the inoculants. The engineering of environments is believed to support resistance to biotic and abiotic stressors, plant fitness, and productivity. Population characterization is crucial in microbiome manipulation, as well as in the identification of potential biofertilizers and biocontrol agents. Next-generation sequencing approaches that identify both culturable and non-culturable microbes associated with the soil and plant microbiome have expanded our knowledge in this area. Additionally, genome editing and multidisciplinary omics methods have provided scientists with a framework to engineer dependable and sustainable microbial communities that support high yield, disease resistance, nutrient cycling, and management of stressors. In this review, we present an overview of the role of beneficial microbes in sustainable agriculture, microbiome engineering, translation of this technology to the field, and the main approaches used by laboratories worldwide to study the plant–soil microbiome. These initiatives are important to the advancement of green technologies in agriculture.

## 1. Introduction

One of the major issues confronting today’s modern agriculture is optimizing sustainable crop output in order to ensure global food security. Furthermore, climate change has exacerbated the impact of environmental stresses such as drought, flooding, heat, and salinity on world food productivity [1]. In addition, the current agricultural practice of utilizing agrochemicals for optimizing yield has resulted in devastating environmental consequences to soil health and fertility [2,3,4]. Hence, to maximize crop output, it is clear that novel methods must be developed and investigated. One of these methods is the incorporation of beneficial microbes into agricultural practices [5,6]. While several studies have reported the role of microbes in plant fitness as well as in soil health and fertility, there is still a need to elucidate the relationship between the microbiome and plant health. Among the aspects of plant–microbe interactions that should be studied are their role in the plant immune responses, signaling pathways (both in plants and microbes), positive and negative interactions between plants and microorganisms, and microbial function in plant productivity [7,8] These findings will enlighten us regarding the entire process of plant–microbe interaction and the discovery of microorganisms that can be exploited to boost crop output in the near future [9,10].

By studying the plant microbiome, we are able to expound on the functional and structural diversities of the microbial communities linked to specific plants and ecosystems. The microbial diversity observed across regions and organs in the phyllosphere, rhizosphere, and endosphere has been well-documented by researchers [11,12]. Plants, in general, use a variety of tactics to favor and support microbial colonization, such as the presence of specialized structures (e.g., hairs, trichomes) or production of secondary metabolites. A comprehensive approach towards deciphering the microbial population and its relationship with plants remains a developing area of research pursued by many laboratories worldwide [11,13]. The diversity in the microbial population is influenced by factors such as host species, selection pressure, environment, developmental stage, and agricultural practices [14]. Several studies have focused on specialized or niche communities to link the population/community diversity with specific stresses or environmental pressures [15].

These observations clearly highlight the need for more thorough and in-depth studies to contribute towards the information and mechanisms that underly the microbiome assembly [16,17]. Technologies for studying microbial diversity and the composition of a specific plant microbiome have advanced significantly. These technologies have moved us from culture-dependent identification, which has its limits in terms of providing a complete picture of the microbiome, to more high-tech methods which achieve higher-resolution images of the microbiome. The development of novel high-throughput techniques and technologies has revealed multitrophic interactions in the black box of plant–microbe interactions [18]. Plant-beneficial microorganisms can now be altered thanks to the advancement of these high-throughput technologies. Microbiome engineering may be an alternate method for understanding, manipulating, and developing corresponding technology for building microbial populations which are critical to plant health and productivity in this scenario [19]. The new and emerging technologies will prove to be useful in deciphering the depth of microbiome diversity in any given ecosystem [20]. Hence, in this review we will address the microbiome in terms of its benefits; its shaping; its response to the environment; plant- and soil-associated microbiomes; and the tools that have been developed to elucidate, understand, and modify plant–microbe interactions.

## 2. Connected Plant Microbiome

Microorganisms naturally colonize soil and plant systems. These plant–microbe interactions are regulated through interrelated chemical signaling. One of these micro-communities, the rhizosphere, harbors microbial diversity that is regulated by the plant root–soil dynamics; it involves root exudates that recruit microbial communities. The rhizosphere also contributes to the development of endophytic communities within the plant. Endophytes cause no negative effects to the plant and are free from environmental control. Finally, the phyllosphere colonizes any of the above-ground plant parts [21,22]. These three micro-communities connect the above- and belowground environments with their respective microbial diversities and communities. These micro-communities have been studied in order to shed some light on their potential for agricultural sustainability, growth, and development [23,24,25].

Beneficial microorganisms found in the plant environment have numerous benefits to the plant ecosystem, including nutrient fixation and solubilization, stress management, phytohormone production, plant phenology, improved yield, and many other positive effects. Previous studies have linked these positive effects to the root exudates/semio-chemicals, which basically function as secondary metabolites with the ability to elicit structural and physiological changes in the rhizosphere [22,23]. The majority of these metabolites are formed through complex pathways, such as polypropanoids, alkaloids, and polyketides. The beneficial interactions between plants and microbes are facilitated by chemical mediators such as terpenoids, flavonoids, and ethylene, which are induced in response to particular triggers [22,26].

These rhizodeposits cause quorum-sensing responses in microbial communities and release a variety of signaling substances, such as antibiotics, carbohydrates, hormones, organic acids, and amino acids, which boost the plant’s defense against attacks and stressors [27]. Jin et al. [28] revealed that root systems are able to secrete chemical compounds that potentially induce chemotactic responses, swarming, and biofilm production. Several reports have concluded that root exudates are versatile in nature and can mitigate both biotic and abiotic stresses by establishing suitable rhizospheric microbiomes [29,30,31,32]. Understanding the chemically regulated process of plant–microbe and microbe–microbe interactions in plants has provided many answers to the black box of plant–microbe interaction and regulation [33,34,35,36,37].

## 3. Microbiomes: Above- and Belowground Connection

Through the latest technologies, such as next generation sequencing (NGS), it has become increasingly possible for the microbial profiles of above- and below-ground communities to be examined at the taxonomic and functional levels [38]. Extensive metagenome profiling has been conducted for commercially viable crops. This is largely due to the exploitation of plant–soil-associated microbes for sustainable agriculture. These studies show that plant microbiomes are predominantly populated by bacteria, viruses, fungi, oomycetes, nematodes, and algae, and bacterial communities such as Proteobacteria, Actinobacteria, and Bacteroidetes remain dominant communities [38]. Unlike the root microbiome, the leaf microbiome is genetically impacted by the host plant, where many leaf microbiome species are shared with root microbiomes, suggesting soil acquisition. All of these studies also highlighted that there are conserved microbial taxa that exist across all plant–soil environments. However, more studies are required in order to comprehend how plant–microbe and microbe–microbe interactions can impact the growth and development of plants [38,39,40]. The microbiome data between studies, however, cannot be compared due to the use of different sampling methods, primers, and sequencing platforms [39,40].

### Factors Affecting the Microbiome

All plant microbiome studies have shown that the structure and profile of the community is determined by the environment. These metagenome studies clearly indicate that biotic and abiotic stresses exert an influence on the predominance of taxa within the community. One important factor that dictates the microbiome’s composition is the genotype of the host. The genotype varies in several aspects, including immunity, secretions, age, morphology, and physiology [41]. This results in the rhizosphere and phyllosphere communities being different among different plant species. Given that the plant genotype determines nutritional quantity, chemical signaling pathways, and other characteristics of the plant, these factors individually or collectively control the exudates, which in turn control the recruitment of microbes to the plant [42]. One example of a genotype differing between varieties was seen in the microbiomes of indica and japonica root [42,43]. The diversity and abundance seen in and around indica show that the number of nitrogen-fixing organisms was especially high in indica as compared to japonica. This positive plant selection pressure results in symbiotic species colonizing the rhizospheric and phyllospheric niches [43]. However, in the event that there are plant–pathogenic interactions or an anthropogenic environment in a particular ecosystem, a shift in the microbiome will occur, resulting in negative consequences for the plant [44]. Further, the geographical factor also influences the microbiome distribution, as there may be differences in the genera and species found to be endemic to certain locations [43,44].

The bacterial and fungal populations in the rhizosphere are significantly influenced by the characteristics of the soil. Microbes are a natural source of nutrients through the various biogeocycles found in the environment. The establishment of microbial communities is influenced by soil characteristics such as pH, moisture content, and soil structure [45,46]. Even when grown in a comparable soil environment, different microbial communities are attracted by different plant species in the rhizosphere, rhizoplane, and endosphere. As opposed to this, where specific microbe populations are related to plant species or genotypes, there is also a population of core plant microbiome which is attracted to the plants regardless of the environment or soil structure. Typically, interactions between a plant’s genetics, related microbiome, and environmental variables will lead to changes in the plant’s phenotype. Overall, the organization of the microbial community is shaped by all of the elements mentioned above, and is acquired vertically through propagation or changes in the environment [47].

Many reviews have focused on how biotic and abiotic stresses influence the plant microbiome. Generally, disease and pests play a crucial role in changing the microbial dynamics in and above the soil. Similarly, abiotic stressors such as drought, flooding, salinity, heat stress, etc., have a profound effect on the plant and soil microbiome. For instance, drought results in loss of water in the plants as well as the soil, which impacts the plant–soil microbiome [48,49]. This results in a drop in certain plant soil microbial taxa, while certain others that help cope with drought stress and water acquisition increase within this stress period. Similarly, in submergence, the soil goes through an anoxic state while the plant tissue goes through stress from submergence in water. This too results in a change in microbial profiles, which results in an increase in microbes that survive in anoxic environments and, at the same time, performs normal functions in the soil to provide protection and nutrients to the plant [48,49].

## 4. Role of Core and Hub Microbiome

Through high-throughput sequencing, the core microbiome that exists within the host was identified. A few bacterial communities may have had a long-standing relationship with plants because of the high overlap of the core microbiome in multiple accessions as well as phylogenetically distinct plants. This relationship extends to the colonization in specific tissues and the stages of development [22,50,51,52,53,54]. Though persistent in the host, they make up only a small portion of the microbiome’s richness, but remain relatively abundant. Some of the core genera that have been reported consistently by researchers are *Sphingomonas*, *Burkholderia*, *Pseudomonas*, *Bradyrhizobium*, *Rhizobium*, and *Mesorhizobium*. These microorganisms are not only widespread and persistent, but also very numerous [51,52,53,55].

Generally, the core microbiome has been linked to plant development and colonization. However, these links have not been established experimentally. While microbes are known to improve plant function, there is no clear indication of co-evolutionary ties between a plant and its microbes [16,56,57]. The location at the cellular/tissue level of these microbes has been studied bioinformatically. Through these studies, a group of “hub” microorganisms has been identified, which are presumed to hold a role in overseeing the network structure and processes at the community level within the microbiome. Therefore, if there is any interruption in the function of a particular community, this disruption can be attributed to the lost or impacted hub community [51,58,59]. Niu et al. [60], in their report, stated that the loss of the hub species *Enterobacter cloacae* resulted in the extinction of the other communities in the ecosystem. Thus, it is necessary to identify hub communities and to determine their functionality in normal and stress conditions.

## 5. Beneficial Microbes for Sustainable Agriculture

### 5.1. Beneficial Microbes and Their Mode of Action

Studies on plant microbiomes have indicated that microbes act on plant hosts either directly or indirectly. The direct influence of the plant microbiome includes nitrogen fixation; phosphate and iron sequestering; hormonal regulation (gibberellic acid (GA), abscisic acid (ABA), cytokinin (CK), and auxin/indole-3-acetic acid (AUX)); and the production of enzymes such as 1-aminocyclopropane-1-carboxylic acid (ACC) deaminase [61]. The reduction in host damage as a result of infection can be achieved through one or more of the following, i.e., production of antibiotics (such as bacteriocins, lipopeptides, proteases, and siderophores); volatile compounds; competitive exclusion; predation; or microbe-mediated immunity, which can activate or suppress innate immune responses in the host. This is the indirect means by which microbes control processes in the host [62].

### 5.2. Plant Growth Promoting Bacteria and Biotic Stressors

The overuse of agrochemicals in agriculture has resulted in negative implications for the environment, impacted human health, and resulted in the deterioration of soil health and fertility. In addition, harmful microbes (*Ralstonia* sp., *Xanthomonas* sp., and *Rhizoctonia* sp.) that cause disease incidence become more rampant when chemicals are overused [63]. Hence, the current trend is moving towards green agricultural practices, where beneficial plant microbes are used to improve crop production and reduce disease incidence. Decades of studies have shown that microbes are able to inhibit disease and also ameliorate abiotic stresses on plants [49,64]. They have been implicated in regulating soil health, maintaining nutrient cycles in terrestrials, and mitigating the negative influences of climate change [4,65]. Together, these traits make beneficial microbes an amenable target for crop yield improvement.

Some important groups of microorganisms that address the above are plant growth-promoting bacteria (PGPB/PGPR) [66,67]. This group of organisms promotes plant health and yield through the production of hormones and enzymes [68]. PGPB include microorganisms that have the ability to control diseases, and have been labeled as biocontrol agents [69]. PGPB inhibit pathogens by lowering iron availability to protect against diseases [70]. Furthermore, advances in plant biotechnology have resulted in the development of resistant crops through molecular breeding or transgenics, with varieties carrying genes resistant towards disease, better nutrient content and uptake, and various other modifications [71]. Transgenics is an environmentally friendly solution to the issue of biotic stressors facing agriculture [72]. The post-genomic era offers open opportunities for the development of superior strains or transgenic lines carrying PGPB genes for both abiotic and biotic stress resistance [73].

### 5.3. Plant Growth-Promoting Bacteria and Abiotic Stressors

Abiotic stressors affect plants’ physiology and metabolism. While some plants are able to acclimatize to stress, others are overcome by it. In the above section, we indicated the role of PGPB in disease control through the secretion of hormones and enzymes. PGPB secrete secondary metabolites that enable the acclimatization of plants to stress [72,74]. Several genera of *Pseudomonas*, *Rhizobium*, *Bacillus*, and *Enterobacter* have been identified as good candidates for abiotic stress management [48]. Microbes are rapidly evolving organisms that adapt to their environments quickly through the production of biofilms and exopolysaccharides (EPS), through the adsorption of osmolytes, or by staying dormant [74]. Under these stresses, plants also produce hormones which help to maintain growth and development [75]. For instance, in response to drought, the *Azospirilium* species develops enhanced levels of ABA [76]. In high-salinity conditions, PGPB have the ability to absorb and store osmolytes in the cell without any negative consequences, as EPS produced by these isolates bind the cations, resulting in microbial cell turgidity [77,78].

### 5.4. The Application of PGPB in Sustainable Agriculture

The future direction of sustainable agriculture has microbes as a primary focus. PGPB and fungi have been identified, isolated, and characterized for application in the field. PGPB have several potential uses, especially in enhancing nutrient acquisition and sequestering [68]. These microorganisms are known to form symbiotic interactions with the plants’ root systems and to extend root growth and increase surface area for better absorption of water and nutrients [79]. Some of these isolates are useful for bioremediation, breakdown of toxic materials, improved growth, and biocontrols [72,80]. Nitrogen-fixing organisms such as *Nitrosomonas* sp., *Nitrobacter* sp., and *Rhizobium* sp. have been reported to increase nitrogen availability for plants, thus leading to increases in the plant yield and biomass [81]. Fungi have been utilized in plant growth and yield promotion, and most have been reported to solubilize phosphate and inhibit pathogen proliferation [82]. The advancement in the omic approaches has flooded us with potential microbes [38] for use in biotic and abiotic stress management [73].

## 6. Microbiome Engineering

The role of the plant genotype in the diversity and phylogeny of the soil microbial population implies that the signature communities in a niche ecosystem can be tailored by both the genotype and the environment [83,84] to provide better host performance. Hence, engineering a rhizospheric system for plants can positively influence the plants [85]. However, in order to engineer the community, the desired impact on the host and environment must be established. The following sections will elaborate further on the role of microbiome engineering in sustainable agriculture.

### 6.1. Rhizosphere Microbiome

The ecto- and endorhizosphere are inhabited by a plethora of macro- and microfauna [86]. Several studies have explored the microbial communities within these environments and determined the association between the microorganisms and the hosts’ root systems [86,87,88]. Most of these plant associations are positive to ensure plant fitness [61,89]. Many studies have reported on root endophytes such as *Rhizobium* spp. and *Brevibacillus* spp. in nodulating and non-nodulating plants, respectively, as these plants have contributed efficiently to nitrogen fixation. Further, through the use of metagenome data, researchers have been able to address the taxonomic diversity in different strains that colonize different plant tissues. The overall microbial communities associated with the host regulate various physiological and biological functions, such as stress management, nutrient uptake, and sequestering. Hence, identifying and mapping the function of microorganisms in each type of plant tissue is necessary in order to understand how the plant–microbe associations boost plant function [90,91]. However, very little is known about the trend of colonization in agricultural crops [92,93].

### 6.2. Rhizosphere Engineering

In engineering the rhizospheric community, there are several questions that need to be addressed. Essentially, we would want to engineer the environment to optimize the benefit to the plant. Thus, how would we positively impact the plant? The obvious response would be for us to improve nutrient cycling, nutrient sequestering, resistance/tolerance to salinity and heavy metals, resistance to pests and diseases, and water-holding capacity. Hence, to engineer the environment, we need to utilize all available tools to decipher the microbiome and its functions. Although we are bombarded with a variety of data, many of the host–microbe interactions still remains obscure. Currently, many products available in the market are from the microbial consortium of PGPB and fungi, such as Bactophospin, which utilizes *Bacillus mucilaginosis* (Russian); Flavobacterin, which uses *Flavobacteria* spp. (Russian); and Mamezo, which uses *Rhizobium* sp. (Japan). In our local context, Malaysia produces MYCOGold, which utilizes 4 genera of AMF [94,95]. However, there have also been PGPB identified in the laboratory and greenhouse trials, which showed much promise, but did not perform well when translated to the field [96,97]. This lack of performance was largely due to the inability of these strains to colonize the environment. Therefore, a crucial factor to bear in mind is that the bioinoculant must be able to form strong and stable associations in the soil and with the plant to ensure lasting positioning in the community and structure within that ecosystem [97,98].

The current omics tools has made it possible for us to make designer changes to the environment and to plants [98,99]. In the years to come, with the advent of more sophisticated tools, we will be able to engineer the environment more efficiently. The procedure, however, will remain the same, i.e., that we will need to understand the mechanism that shapes the environment and imitate the symbiotic relationships that exist between the soil, microbes, and plants in nature by engineering identical environments in the field. Once this is accomplished, the bioinoculant will flourish in the seeded ecosystem.

### 6.3. Shaping the Microbiome

In shaping the microbiome, effort needs to be focused on isolating and identifying microbes and their functionality in the rhizosphere. Therefore, there needs to be a database of microorganisms and their specific contributions to the rhizosphere so that, in developing agricultural applications, a more astute decision may be made regarding formulating the consortium [100]. Plant–microbe symbiotic associations should be made an important part of this database, and organisms conducting specific functions should be grouped accordingly. For instance, from previous studies, it has been reported that rhizobacteria such as *Rhizobium*, *Bradyrhizobium*, *Mesorhizobium*, *Azospirilium*, *Azotobacter*, and *Acetobacter* are required for nitrogen fixation [100,101]. In addition, the presence of phosphate-solubilizing and siderophore-producing bacteria increases the nutrient availability for the plant. Further, there is another group of organisms which produces antimicrobials that are important in inhibiting soil pathogens. These organisms exude antibacterial compounds such as 2,4-diacetylphloroglucinol (DAPG), hydrogen cyanide (HCN), oligomycin, bacteriocine, and antifungals [96,102]. Additionally, research has shown that inoculating plants with consortia of plant growth-promoting microorganisms and arbuscular mycorrhizal (AM) fungi aids against biotic and abiotic stresses by producing defense-related chemicals [97,103,104,105,106].

**Figure 1:**
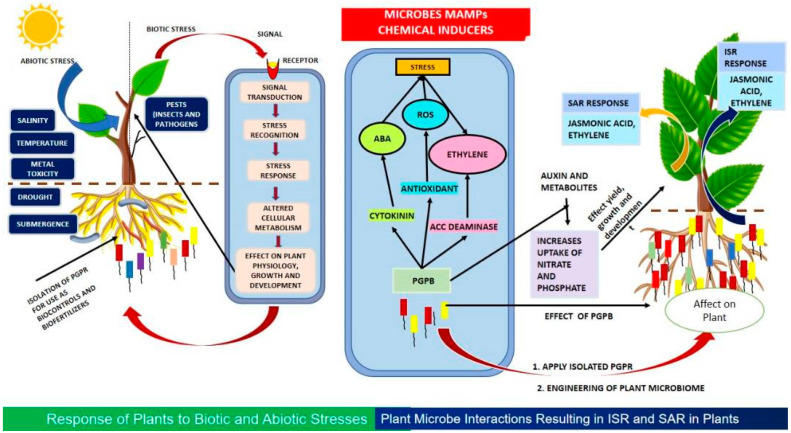
Interactions between plants and microorganisms in biotic and abiotic interactions. This diagram shows how abiotic and biotic stresses can be perceived by the plant, causing changes in cellular metabolism and affecting plant physiology, growth, and development. The stress management in plants, which is elicited by these stresses, is managed through crosstalk and interplay of hormones. In addition, there is crosstalk between signal molecules such as jasmonic acid and salicylic acid that regulate SAR and ISR, respectively. Both of these mechanisms induce a defense response in plants. On the left, we see how abiotic and biotic stresses affects plant physiology, growth, and development. These interactions result in negative impacts on susceptible varieties. Reactive oxygen species (ROS) are produced in response to these stresses. However, plants do possess mechanisms of homeostasis that keep the ROS at levels non-detrimental to the plant. On the right, we have the ISR and SAR, which are produced in response to crosstalk between signal molecules and hormones in plants. The SAR and ISR are instrumental in the defense and response of plants against current and future infiltration or associations by microorganisms, respectively.

Further, the growth and development of plants is also dependent on phytohormones, which are necessary for plant–microbe interactions [107]. Microbial communities create a number of phytohormones, including gibberellins (GA), auxins or indole-3-acetic acid (IAA), and cytokinins. The major role of phytohormones in plant growth and development has been proven through transcriptome studies. The crosstalk between these chemical and signal molecules, such as jasmonic acid and salicylic acid, are crucial in inducing systemic acquired resistance (SAR); they induce systemic resistance (ISR) in plants. Plants can develop resistance to a wide spectrum of pathogens both under- and aboveground by being inoculated with non-pathogenic bacteria. This ISR primarily relies on ethylene and jasmonate signaling pathways. Through this, plants are able to respond more rapidly to the onslaught of pathogens. ISR has been reported in response to various microorganisms, the microbe-associated molecular patterns (MAMPs) of which, including cell envelope components, flagella, and siderophores, activate ISR [24,108,109]. It is interesting to note that some PGPB cause ISR reactions and stimulate plant development by emitting volatile organic compounds (VOCs) [110,111]. A number of *Bacillus*, *Pseudomonas*, *Serratia*, *Azozpirilium*, and *Trichoderma* species are well-known ISR-inducing microorganisms (Figure 1).

Knowledge of how these organisms are regulated and what the active compounds produced by these organisms are is essential information that is required to shape a microbiome. Hence, the database mentioned above is a necessary and detailed description of functionality. The exuded chemical compounds will make the identification of key microbes for specific functions in growth, development, and biotic and abiotic stress management more precise and efficient [74,112].

### 6.4. Rules That Govern Microbiome Engineering

Understanding the ecological mechanisms that control the emergence, persistence, and regional adaptability of the plant-associated microbiome is essential for the successful field application of microbiome therapies. However, up to this point, the majority of the microbiome investigations have concentrated on questions such as “what is in there?”, “what are their functions?”, and “how do they interact with environment and plant?”. We have yet to arrive at a conceptual framework that enables us to comprehend how ecological processes control the microbiome’s assembly and function [113]. For the purpose of creating models that favor successful colonization, proper understanding of the ecological processes that produce and sustain different plant-associated microbiomes is essential. According to the ecological [114] and meta-community theories [115], multi-species assemblages are the result of the interaction between four main co-evolutionary processes in microbiome–host interactions. These are dispersal, diversification, selection, and drift. Among these four processes, dispersal enriches the process of diversification. Meanwhile, drift and selection contribute towards the relative abundance of the microbial species. There exists a complex interaction between these mechanisms and the ecological traits of microbial communities (such as resistance, resilience, and functional redundancy), which are important in preserving community stability and formation.

In the late stages of plant development, selection plays a role in community assembly, while dispersal and drift become significant in the seed and root developmental stages [116]. At different stages of development, the processes shift in relative importance. The assembly and performance of plant-associated microbiomes can be impacted by the arrival order, which is referred to as priority effects [16]. By increasing the number of suppressive bacteria or by activating the plant immune system, uncommon taxa, for instance, may have a priority effect on the microbiome during different stages of plant development [117]. The significance of priority effects for structuring the composition of the microbial communities can be studied using SynCom. Findings from these studies indicate that the founding taxa have a long-lasting impact on the formation of microbial communities, and are, thus, resistant to invasion by newcomers.

Therefore, based on the above conclusions, we may conclude that microbiome inoculation can be effective when applied in the early stages of development. Local scale community assembly can be impacted by abiotic stresses, migration from other ecosystems, community structure, and internal ecological interactions [115]. Microbial communities should be considered as dynamic, ever-changing systems that warrant careful study. Additionally, studies focusing on arbuscular mycorrhizal plants have offered evidence that genetic variations of isolates influence their effects on host–microbe interactions and, thus, affect their potential in influencing host–plant fitness [118,119].

### 6.5. Shaping the Microbiome through Plant-Mediated Strategies

When addressing plant-mediated strategies, two distinct methods—plant breeding and genetic engineering—are used to control plant characteristics. Using plant breeding techniques to determine the microbial community is of interest for boosting crop yield and resilience [120]. Microbiome selection has been incorporated into plant breeding programs, focusing on specific taxa and functions that can produce good yields. Transgenic plants with increased resistance to disease and soil physicochemical tolerance (phosphate-limited soils) have improved disease suppression as well as ability of plants to grow on acidic soils [121,122]. Further, transgenic tobacco and Arabidopsis plants have produced exudates which altered the rhizospheric pH to acidic, thus improving the plants’ resistance, growth, and ability to mineralize nutrients from the soil [123,124].

According to the research conducted by Ellouze et al. [125], certain chickpea cultivars attract a more advantageous group of microorganisms that shape the microbiome as well as increase the yield potential of durum wheat. Numerous studies have been conducted to regulate plants by altering the synthesis of crucial exudates that govern the formation of particular plant–microbiome interactions. However, the importance of breeding or molecular breeding with emphasis on reshaping the microbiome have not received as much attention. Plant-mediated strategies regarding the rhizosphere microbiome are currently being addressed using molecular- and genome-based techniques that are more precise and informative. These techniques will be elaborated on in Section 8.

## 7. Translation of Knowledge to Field Application

All works conducted in the discovery phase must be translated into implementation to improve plant growth and development under all types of environmental pressure. Hence, there needs to be technology development, translational plans, and policies developed to ensure translation from the laboratory to the field. In this section and the following, we will look into translational technologies to support this transition.

### 7.1. Engineering a Host-Mediated Microbiome for Sustainable Agriculture

The successful implementation of microbial-based solutions into the enhancement of plant performance depends on the capacity to develop communities with beneficial traits. Due to their superior ability to acclimatize to various environments, soil types, and/or plant niches, native microbiota have a higher likelihood of developing and expressing favorable traits [126]. Hence, it is possible to select for a robust, plant-optimized microbiome that is resistant to random invasion utilizing experimental evolutionary techniques [127]. Beneficial microbiomes that are optimized for a particular environment can remain in, on, and around plants for many generations [128].

Host-mediated microbiome engineering utilizes the knowledge of microbial communities adapted to the host to accomplish specific functionalities [18,129]. For instance, by artificial selection, specialized microbial communities can be built up that alter plant traits in a highly reproducible manner [130]. In order to maximize their chance of reproducing under various stresses, plants have evolved a method known as accelerated flowering time [131]. In this case, altering the plant microbiome artificially leads to the development of stress-tolerant phenotypes with improved productivity in response to volatile climatic conditions [132].

### 7.2. Engineering Plant Microbiomes for Sustainable Agriculture

The results of meta-analyses have revealed that domestication has caused taxonomic alterations in root microbial communities, highlighting the importance of understanding the function of any missing microorganisms that can give wild species a competitive advantage over their domesticated counterparts [133]. The heredity of microbiomes and their connection to agronomic traits are both supported by recent studies. Studies conducted on different genotypes of canola showed that there was a notable difference in the percentage of microbial species [134,135]. As there is growing evidence that the microbiome affects host performance, breeding programs must successfully integrate genotype, environment, microbiome, and management interactions [136]. Therefore, to identify plant genes with real impact on the microbiome, multi-year field and location studies must be conducted to identify connections between the genotype, environment, and microbes. The connection between the three is essential for the sustainable translation of beneficial microbes to the field [43,137].

Genome-wide association studies (GWAS) have shown that plant genes play a role in plant-associated microbiome assembly. The abundance of particular rhizosphere microbiome subgroups is strongly connected with the presence of these genes, which are shared by diverse plant types [138,139]. By modifying these potential genes, it is possible to produce designer plants with particular microbiomes and advantages. For instance, genes implicated in stress responses and glucose metabolism have an impact on the phyllosphere microbiome of rice [129]. The phyllosphere microbiome undergoes changes in the overexpressing line that are related to structural changes and induced immune responses [140]. The genetic basis of interactions between the microbiome and the host can be better understood by GWAS, but these may also reflect false connections and are not always capable of establishing causal relationships [141]. By affecting the expression of various genes involved in the production of plant hormones, the resultant assemblage of soil microbiomes affects the plant’s physiology [142]. Altered chemical signals in rhizobium–legume symbiosis have changed how plants are able to preferentially attract nitrogen-fixing rhizobia [143]. In addition, plant hormones such as salicylic acid [144] or secondary metabolites such as coumarin are known to shape the root microbiota under stress, and may be utilized to genetically engineer crops for sustainable agriculture.

### 7.3. Management Practices That Optimize the Microbiome

While small amounts of conducive and disease-suppressing soils can be combined to transfer disease-suppressive properties, disease-suppressing soils can also be created through specific management techniques, such as tillage and crop rotation [145,146]. It is possible to steer agroecosystems in healthier directions by adopting the advantageous microbial impacts of some management practices. For example, the intercropping of leguminous crops with non-legumes can result in gaseous signal exchange between plant-associated microbiomes [29]. Overall, it has been reported that intercropping enriches soil microbiota, resulting in better fitness and yield. The impacts of organic farming and conservation tillage on the soil microbiome result in increased soil nutrient cycling, which leads to good growth and yield [16,147]. The use of fertilizers and machinery, site conditions, profitability, and crop varieties are only a few variables that influence the overall management decisions and practices that influence the optimization of the microbiome. Further studies must be conducted to optimize the use of management practices and microbiome engineering for the purpose of positively impacting the agricultural industry.

### 7.4. Optimized of Microbiomes through Genetical Engineering

Genome-based methods have uncovered a large number of genes in microbes that mediate plant–microbe interactions [148]. The creation of superior microbial inoculants has a great deal of promise thanks to the engineering of microbial genes that promote advantageous features. Recently, the symbiotic *Snodgrassella alvi* was genetically modified via RNA interference (RNAi) [149] to shield honeybees from viral infection and parasite mites. Using similar techniques, it is possible to produce bacterial endophytes that cause plants to react defensively to pathogens and pests. The creation of genome-editing tools, such as CRISPR and CRISPR/Cas 9, has enabled the modification of genes and genomes to achieve advantageous plant- and microbe-associated traits. This technology, according to Farrar et al. [9], allows for the construction of improved and enhanced traits by influencing the regulation of genes/genomes. According to Goold et al. [150], significant work is being carried out to rebuild microbial circuitry (new gene networks created using synthetic biology methods) and biological sensors that may be utilized in agriculture. A genetically engineered strain of *Pseudomonas putida* called BananaGuard has been created to combat the *Fusarium oxysporum* that causes Panama disease in banana. This altered bacteria detects fusaric acid produced by *F. oxysporum* and produces inhibitors of fungal growth. The system is inbuilt with a kill-switch that triggers self-destruction of the inhibitors when the fungus is no longer found [151].

Recent advances in nitrogen fixation have become possible through the engineering of naturally existing endophytic or epiphytic bacteria of cereal crops [152]. N_2_-fixing strains have been further genetically modified such that they would express N_2_-fixing genes in response to the presence of naturally existing chemical signals in plant-associated microbiomes. Targeted genome editing has advanced to the point that it is now conceivable to develop designer genetic microbial circuits that respond to signals from plants to maximize host–microbe interactions [153]. Although there are some regulatory uncertainties within some jurisdictions, gene editing will remain a frontier science with immense potential for plant–microbiome associations. Currently, there is some work being conducted that aims to develop microbiomes with altered nitrogen-fixing abilities to successfully replace chemical fertilizers in the field [150]. Inoculating natural systems with non-indigenous microbial strains, however, requires much research to determine that there are no detrimental effects on the diversity of native plant and microbial species, and it is essential that all of these processes are conducted in accordance with policies and jurisdictions regarding the application of foreign organisms into the field [154,155].

## 8. Methods of Microbiome Studies

Methods must be developed that can provide us with data to make an informed and educated selection of suitable candidates for the development of biofertilizers, biocontrols, etc. The following are some of the most commonly used methods for above- and belowground microbial community identification, which involves organisms in and on plants, as well as microorganisms in the soil.

### 8.1. CRISPR/Cas9

CRISPR/Cas9 is a new, emerging technology for addressing plant–microbe interactions [156]. In addition, CRISPR/Cas9 has been used to genetically modify microorganisms to increase their beneficial effects on crops [157]. *Alternaria alternata*, *Colletotrichum sansevieriae*, *Fusarium proliferatum*, *Phytophthora* spp., and *Sclerotinia sclerotiorum* are only a few of the phytopathogens that have been studied using the CRISPR/Cas9 system [156,158]. Fusarium specifically causes a number of plant diseases by producing mycotoxins such as fumonisins. The polyketide synthase gene *FUM1* in Fusarium is in charge of producing fumonisins. The *FUM1* was deactivated using the CRISPR-Cas9 method, and the altered mutants failed to make fumonisins [159]. Through the creation of CRISPR-Cas9 endogenous gene tagging, the infection process of fungal diseases could be elucidated. It is possible to research the subcellular localization of fungal proteins through these endogenous fluorescent tagged genes [98]. Another example of editing is shown in *Trichorderma atroviride*, namely, the *ace1* gene, which enhances the production of polyketide biosynthesis genes, heightening its control over certain soil pathogen diseases [160]. As a result, the activation of gene clusters increased the potential of these microorganisms to be used as biocontrols. Hence, this method could uncover brand-new ways in which genes and pathways could be enhanced in microbes to provide better control of biotic and abiotic stresses.

**Figure 2:**
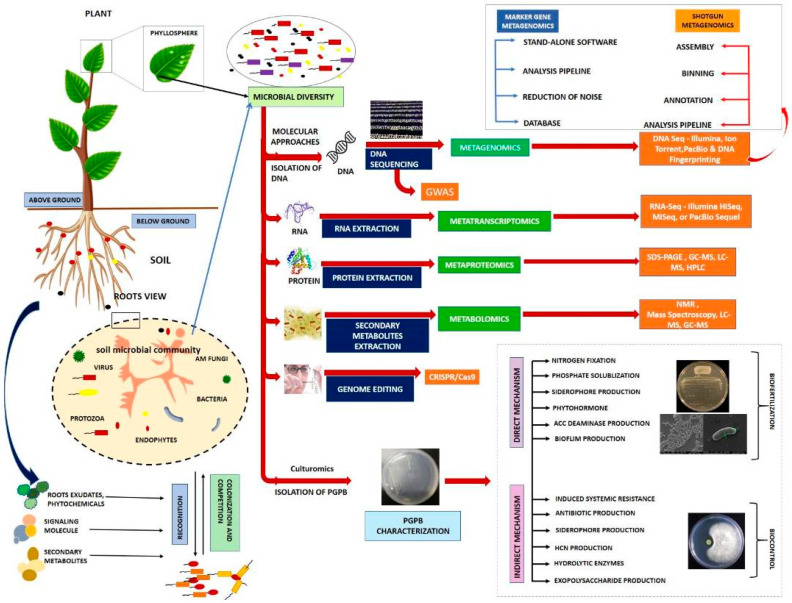
A schematic diagram of several culture-dependent and non-culture-dependent methods that have been utilized extensively in the study of plant–soil microbiomes. The methods listed for the four omics technologies are the most commonly used techniques. The direct and indirect characterization of PGPB is also provided in this diagram. Legend: NMR—nuclear magnetic resonance; LC-MS—liquid chromatography–mass spectrometry; GC-MS—gas chromatography–mass spectrometry; RNA-Seq—RNA sequencing; DNA Seq—DNA sequencing.

In addition, CRISPR-Cas9 has been utilized to modify beneficial microorganisms by improving their functionality. It has also been applied to study processes connected to the soil microbiome, such as nitrification and the breakdown of lignocellulose. The nitrate transporter in the rice gene *NRT1.1 B* controls the root microbiota in indica cultivars [142,156]. The efficiency of nitrogen consumption in japonica rice was considerably improved by targeted manipulation of this gene [161]. Two other genes (*OSH15*, *OsAt10*) modified plant cell wall constituents in order to increase saccharification [156,162]. CRISPR elements are naturally present in microorganisms [163] against foreign elements such as bacteriophages. Additionally, it has been demonstrated that polar soil contains more CRISPR genes than tropical soil, which explains the higher disease incidence observed in the tropics [156]. Further studies have also shown that genome editing using integrative plasmids reduces horizontal gene transfers considerably [164]. Therefore, having CRISPR repetitions may give plant-beneficial bacteria an evolutionary edge for improved adaptation (Figure 2).

### 8.2. Genome-Wide Association Studies (GWAS)

Numerous studies on the relationships between plants and microbes have used GWAS to investigate the relationship between the plant genotype and microbial recruitment [165,166]. GWAS was used to identify loci associated with blast resistance in rice [167,168]. A single-nucleotide polymorphism (SNP) chip of African rice cultivars identified genomic areas connected to rice blast resistance [169]. Further, the effectiveness of GWAS in identifying disease resistance was also observed in maize [166,170]. Recent studies have also shown the ability of GWAS to identify the relationship between leguminous plants and their nodulating bacteria [171]. Horton et al. [172] employed a panel of 196 *Arabidopsis thaliana* genotypes to correlate the leaf microbial population through taxonomic marker gene sequencing of the bacterial or fungal populations. Through this study, they found that there was an additive genetic variety association between the host and the microbial community in the leaves and roots. With the help of these limited community datasets, Horton et al. [172] were able to successfully correlate host SNPs to community-level characteristics of the most common individual taxa that are connected to the leaf microbiota. To date, not many studies have been conducted using GWAS to study the microbiome relationships above- and belowground. However, GWAS have been used extensively in human–microbiome interaction, proving to be a potentially useful tool for association studies of plant–microbe interactions [173,174,175] (Figure 2).

### 8.3. Microbiome Sequencing Platforms

The two primary strategies utilized in metagenomic research to target the enormous diversity of environmental investigations are targeted and shotgun sequencing. Since these methods enable researchers to expedite the entire process at a lower cost, the Illumina platform is the choice method for metagenomic sequencing. It provides many millions of brief, incredibly accurate random reads that can be combined or utilized as markers for certain metabolic pathways and/or groups of microorganisms. Although other cutting-edge platforms, such as Ion Torrent and PacBio, have also been created, the Illumina platform still remains the most effective sequencing platform [173,174,176].

In recent years, many PGPB with various phenotypic traits connected to plant growth capacity have been characterized using whole-genome sequencing. For instance, *Brevibacterium frigoritolerans* ZB201705, isolated from salt and the drought stress rhizosphere of maize, is able to manufacture a large number of proteins [177] from a complete genome. This shows that *B. frigoritolerans* ZB201705 could be employed as an inoculant to boost crop yields, even in the presence of abiotic challenges. Pyrosequencing has also been used to elucidate the genome sequence of *Pseudomonas* sp. [178]. Through this study, Pseudomonas was shown to produce chemicals such as siderophores, phosphate solubilizers, ACC deaminase, and indole acetic acid, which contribute to plant growth and development (Figure 2).

There are numerous molecular DNA fingerprinting methods which can be used to examine the endophytic population that inhabits plant tissues, including amplified rDNA restriction analysis (ARDRA), denaturing gradient gel electrophoresis (DGGE), temperature gradient gel electrophoresis (TGGE), and terminal restriction fragment length polymorphism (T-RFLP) [179]. To acquire community fingerprinting, alternative methods such as automated ribosomal intergenic spacer analysis (ARISA), which analyzes the extremely variable area between the 16S and 23S rDNA, can also be used [179]. However, as of now, metagenomics techniques utilizing NGS have largely surpassed the development of all these DNA fingerprinting techniques [180]. In an effort to better understand the potential advantages of endophytes for the host plant, efforts have been made to sequence entire genomes of over twenty genera of endophytes isolated from various hosts [181].

### 8.4. Metatranscriptomics, Metaproteomics, and Metabolomics for Understanding the Microbiomes

Through rhizosphere microbiome metatranscriptomics of wheat, oat, and pea, kingdom-level variations have been identified between these plant systems [182]. Stress (drought stress) also influences the microbiome associated with the root, hence causing it to demonstrate increased transcriptional activity of genes related to amino acids and glucose metabolism [183]. Microbial communities have been more recently developed as biosensors using metagenomic and metatranscriptomic profiling [184]. The development of proteomic and metabolomic approaches to supplement transcriptome data is a result of the poor correlation between the transcriptional and translational processes. With the use of metaproteomic analysis, a thorough understanding of the molecular phenotypes of microbial communities in agricultural plants’ rhizospheres and phyllospheres has been attained [185]. (Figure 2).

These studies have shown notable stability in the dominant microbiomes of organisms and proteins associated with host plants and the environment [89]. Metaproteomics-based inferences of the physiology of the microbiomes also revealed metabolic pathways that permit specialized colonization and adaption in the rhizosphere compartments [89,130,186]. Although microbiome science is still in its infancy, the metabolome has been frequently utilized for identifying plant diseases and causative agents [185]. According to early research [185], the phyllosphere metabolome is altered by the rhizosphere microbiome, and these alterations are related to distinct insect feeding strategies. Studies have also shown that alterations to the root metabolome determine the recruitment of specialized microbial communities that colonize the rhizobiome and plant, affecting the plant’s performance and its interactions with pests and diseases [187,188]. For the detection and measurement of small chemicals that drive plant–microbiome communication and interactions, metabolome information is essential [130,185]. Recent advances in multiomics and integrated informatics have uncovered the complex relationship between plant traits, metabolites, microbes, and minerals in an agroecosystem. We believe that improved sample preparation; extraction techniques; database compilation of proteins, metabolites, and genes; and the development of computational tools to support bioinformatic analyses of big data will advance this field to fully realize the potential of the omic approaches in deciphering the genotype–phenotype relationships [189,190].

### 8.5. Culturomics to Support the Molecular Techniques

The use of microbiomes to produce commercial inoculants requires the use of cultivated bacteria [191]. Microbial cultures are also required for us to progress into omic studies and, at the same time, provide bacterial reference genome sequences for functional studies [192,193]. Many agricultural research universities and institutions maintain culture collections that are related to plant-associated microbiomes. Huge collections of isolates have been subjected to comparative genomics, through which several important genes have been identified. The validation of these genes through molecular techniques is possible due to the availability of cultured members [194]. Despite recent advances in culturomics, genomic diversity in cultured bacterial isolates is still far from reaching saturation [195]. Future studies into the putative functions of plant-associated microorganisms will require impartial culture sequence collections obtained from a wide range of plants and soil types [196]. These collections must also include mechanisms to curate, share, and standardize metadata for the strains contained within them (Figure 2).

In addition to supporting omics platforms with cultures, it is also possible to analyze the direct and indirect mechanisms of plant growth-promoting bacteria using these cultures. According to Glick [197], PGPB can stimulate plant growth both directly and indirectly. Direct methods are those that make use of bacterial features that directly promote plant development, such as nitrogen fixation, siderophores synthesis, ACC deaminase activity, phytohormone generation, biofilm production, and phosphate solubilization. Some PGPB can fix nitrogen from the atmosphere and transform it into a form that plants can use, encouraging plant growth [198,199]. In a study by Sarkar [200], culturomics was utilized to assess the capability of drought-tolerant *Pseudomonas* for nitrogen fixation. In addition, an essential form of PGPB such as siderophilic bacteria can release siderophores to chelate Fe^3+^ in the soil for plant development [201]. By utilizing culturomics, Flores-Felix et al. [202] revealed that the PGPB had the ability to promote blueberry development by dissolving dicalcium phosphate to form siderophores. PGPBs also generate ACC deaminase, an enzyme that reduces ethylene levels in plants to prevent ethylene-induced suppression of root growth and trigger stress reactions [203,204,205]. Culturomics was also applied by Chicca et al. [206], who demonstrated that PGPB (*Microbacterium*, *Achromobacter*, and *Pseudomonas* spp.) produced ACC deaminase activity, which promotes plant development. Further, phytohormones including auxins, cytokinins, and gibberellins, which control different aspects of plant growth and development, can also be produced by PGPB [207]. A study by Sahu et al. [208] combining metabarcoding and culturomics methods in profiling the rice phyllosphere microbiome identified extensive and useful microbial groups for blast disease mitigation. The results also demonstrated increased expression of genes related to defense, including *OsCERK1* and *OsCEBiP*, as well as genes related to phytohormones, including *OsFMO*, *OsPDF2.2*, *OsNPR1*, *OsPR1.1*, *OsEDS1*, and *OsPAD4*. PGPB also had the ability to create biofilms, which are microbial communities that adhere to surfaces and promote improved colonization, nutrition substitution, and adaptation to stress [209]. PGPB also have the capacity to solubilize phosphate and increase its availability for absorption and use by plants. By utilizing culturomics, Flores-Duarte et al. [210] identified 13 features in PGPB, including biofilm development and phosphate solubilization, in their study.

The indirect mechanisms of PGPB action include the production of exopolysaccharides (EPS), hydrolytic enzymes, hydrogen cyanide (HCN), ISR, antimicrobial compounds, quorum quenching, competition, and siderophores. The production of EPS by PGPB can improve soil aggregation, provide pathogen protection, and encourage nutrient and water retention [211]. Furthermore, the production of HCN by PGPB has antibacterial properties and can halt the growth of plant diseases [212]. Additionally, many PGPB have secretion systems that enable them to create antimicrobial substances, including antibiotics, organic volatile compounds, and lytic enzymes, which can limit the development of potentially phytopathogenic microbes [213]. PGPB also support plant resistance by inducing systemic resistance to a variety of diseases and initiating the plant’s defense mechanisms [214,215]; they secrete hydrolytic enzymes, including chitinases, proteases, and cellulases, that break down different parts of the pathogen’s cellular wall or extracellular structure [216]. Combining a culturomics approach with molecular methods would help to characterize the indirect and direct mechanisms of PGPB.

## 9. Conclusions

In the process of moving towards sustainable agriculture, agricultural practices must reduce the extensive usage of agrochemicals. In this review, we have presented the potential of beneficial microbes to enhance growth, development, and disease suppression in the field. However, the effectiveness of beneficial microbes in field applications has been less than satisfactory. This is largely due to the fact that the newly introduced microbes must thrive in the environment and maintain a steady and stable community for the benefits to be harnessed. This has brought forth technologies such as sequencing and multiomics platforms, which have enabled us to visualize, to some extent, the diversity, communities, and structures of microorganisms in any given environment. This has created opportunities for technologies such as microbial engineering to offer designer solutions for specific environments thus achieving greater efficiency and sustainability. This technique promises answers to various gaps in knowledge, such as providing the right microbial consortia based on the plant species and soil environment to ensure proper recognition and colonization of the soil and roots by the inoculants. In this regard, the advancement of “microbiome-driven cropping systems” may herald the next agricultural revolution and a more sustainable method of plant production. Furthermore, the development of modified crops or organisms may yield the desired advancement towards zero hunger for the continuously expanding human population. This will be made possible by the application of multiomics approaches combined with genome editing techniques such as CRISPR for improving nutritional status, disease resistance, and crop yield.

In the years to come, however, there is much work that needs to be conducted to comprehend the genetics and engineering of the intricacies behind the ecological and metabolic networks that govern plant-associated microbe interactions. For instance, research needs to go beyond the identification of causative or beneficial organisms into “how” this information may be used reproducibly to enhance plant growth and development and to reduce disease incidence and spread. We also need to reduce wastage and redundancies in research by producing standardized techniques and a center for the collation and annotation of meta-data. These techniques must be made such that they can be utilized by any laboratory worldwide for the translation of laboratory-raised products to the field. We also need to focus on developing new technologies that are more efficient, accurate, cost-effective, and quick to use both in the laboratory and in the field. The technologies that are developed should not be restrictive to only the well-funded research groups, but should be attainable for small laboratories. Bioinformaticians need to continuously come up with new software or upgraded versions of the existing platforms to increase the depth, speed, and quantity of information harnessed from the data. The upgrade does not stop with the equipment and the techniques; the upskilling of researchers needs to evolve along with trends in the field of plant-associated microbiome research. This field has a bountiful future, and there is room to push the boundaries of knowledge and technologies further.
